# Association of obesity with prostate cancer: a case-control study within the population-based PSA testing phase of the ProtecT study

**DOI:** 10.1038/sj.bjc.6606066

**Published:** 2011-01-25

**Authors:** P Dimitropoulou, R M Martin, E L Turner, J A Lane, R Gilbert, M Davis, J L Donovan, F C Hamdy, D E Neal

**Affiliations:** 1 Department of Oncology, University of Cambridge, Addenbrooke's Hospital, Box 279 (S4), Cambridge CB2 0QQ, UK; 2 School of Social and Community Medicine, University of Bristol, Canynge Hall, 39 Whatley Road, Bristol BS8 2PS, UK; 3 Nuffield Department of Surgery, Faculty of Medical Science, University of Oxford, John Radcliffe Hospital, Oxford, UK

**Keywords:** prostate cancer, case–control study, obesity

## Abstract

**Background:**

Obesity has been inconsistently linked to prostate cancer, mainly with mortality rather than incidence. Few large-scale studies exist assessing obesity in relation to prostate-specific antigen (PSA)-detected prostate cancer.

**Methods:**

We used cases and stratum-matched controls from the population-based PSA-testing phase of the Prostate testing for cancer and Treatment study to examine the hypothesis that obesity as measured by body mass index (BMI), waist circumference and waist-to-hip ratio (WHR) is associated with increased prostate cancer risk, and with higher tumour stage and grade. In all, 2167 eligible cases and 11 638 randomly selected eligible controls with PSA values were recruited between 2001 and 2008. A maximum of 960 cases and 4156 controls had measurement data, and also complete data on age and family history, and were included in the final analysis. BMI was categorised as <25.0, 25.0–29.9, ⩾30.0 in kg m^−2^.

**Results:**

Following adjustment for age and family history of prostate cancer, we found little evidence that BMI was associated with total prostate cancer (odds ratio (OR): 0.83, 95% confidence interval (CI): 0.67, 1.03; highest *vs* lowest tertile; *P*-trend 0.1). A weak inverse association was evident for low-grade (OR: 0.76, 95% CI: 0.59, 0.97; highest *vs* lowest tertile; *P*-trend 0.045) prostate cancer. We found no association of either waist circumference (OR: 0.94, 95% CI: 0.80, 1.12; highest *vs* lowest tertile) or waist-to-hip ratio (WHR; OR: 0.93, 95% CI: 0.77, 1.11; highest *vs* lowest tertile) with total prostate cancer, and in analyses stratified by disease stage (all *P*-trend>0.35) or grade (all *P*-trend>0.16).

**Conclusion:**

General adiposity, as measured by BMI, was associated with a decreased risk of low-grade PSA-detected prostate cancer. However, effects were small and the confidence intervals had limits very close to one. Abdominal obesity (as measured by WHR/waist circumference) was not associated with PSA-detected prostate cancer.

Obesity is associated with a number of chronic diseases, including coronary artery disease, hypertension, diabetes and some cancers (Rodriguez *et al*, 2001; Calle *et al*, 2003). The association of obesity with hormone-related cancers, such as prostate cancer, has been inconsistent in epidemiological studies. Obesity has been more strongly positively associated with prostate cancer mortality rather than showing consistent associations with incidence (Andersson *et al*, 1997; Rodriguez *et al*, 2001; Calle *et al*, 2003; Wright *et al*, 2007). Studies examining obesity during prostate development in earlier life, and its possible effect on prostate cancer development later, have also produced contradictory results (Giovannucci *et al*, 1997; Schuurman *et al*, 2000). Overall, there are studies showing positive (Gronberg *et al*, 1996; Veierod *et al*, 1997; Putnam *et al*, 2000), null (Whittemore *et al*, 1995; Giovannucci *et al*, 1997; Nilsen and Vatten, 1999; Habel *et al*, 2000; Lee *et al*, 2001; Jonsson *et al*, 2003; Gallina *et al*, 2007) and inverse (Giovannucci *et al*, 2003; Wright *et al*, 2007) associations between body mass index (BMI) and prostate cancer risk.

The inconsistency between these studies might be attributed to a possible interaction between obesity and factors such as age, or to differential effects of obesity on low-grade and high-grade cancer implying aetiological heterogeneity for different tumour subtypes (Freedland *et al*, 2006). In addition, many studies do not distinguish between central and peripheral adiposity.

There is some evidence, from prospective studies, that obesity is associated with a reduction in risk of incident prostate cancer (Giovannucci *et al*, 2003; Wright *et al*, 2007). There are biological mechanisms which may explain potential protective effects against initiation of prostate cancer (Giovannucci *et al*, 2003). For example, there tend to be lower circulating levels of testosterone in obese compared with non-obese men (Pasquali *et al*, 1991; Field *et al*, 1994). Observed protective effects may also be an artefact of haemodilution of prostate-specific antigen (PSA) values in larger men, thereby reducing PSA threshold-based detection of true cancers by misclassifying them as non-cancers (Grubb *et al*, 2009).

In two case–control studies (Hsing *et al*, 2000; von Hafe *et al*, 2004), central adiposity was associated with an increased risk of prostate cancer progression. However, the risk was increased for non-advanced stage as well. Most central adiposity case–control and prospective studies did not show any associations when BMI was assessed (Hsing *et al*, 2000; Lee *et al*, 2001; von Hafe *et al*, 2004) and Giovannucci *et al* (1997), in their prospective cohort study, showed no association between either adult BMI or waist-to-hip ratio (WHR) and risk of total or advanced prostate cancer (Giovannucci *et al*, 1997). One prospective cohort study (MacInnis *et al*, 2003) found no overall association with prostate cancer, but modest associations with the risk of aggressive disease, whereas other studies of the same type (Hubbard *et al*, 2004) report an overall increased risk with increasing WHR.

We conducted a case–control study nested within the PSA-testing phase of the Prostate testing for cancer and Treatment (ProtecT) study (Donovan *et al*, 2002) to examine associations of obesity with screen-detected prostate cancer. The measures of obesity included BMI, waist circumference and WHR. Associations with obesity were examined for total prostate cancer as well as its subtypes (localised, advanced; high-grade, intermediate grade and low grade).

## Participants and methods

The methodology of the ProtecT study has been described previously (Donovan *et al*, 2002). ProtecT is an ongoing randomized controlled trial that will compare the effectiveness and acceptability of treatments for localised prostate cancer in men aged 50–69 years. Between 2001 and 2008, over 110 000 men aged 50–69 years, from ∼300 primary care centres (general practises) across the United Kingdom, attended prostate check clinics, where histologically confirmed prostate cancer cases were identified through a combination PSA testing, digital rectal examination (DRE) and 10-core transrectal ultrasound-guided biopsy (the latter two investigations only apply to those with PSA ⩾3). Repeat biopsies were offered to men with a normal initial biopsy, in whom there was a high index of clinical suspicion (evidence of high-grade prostatic intraepithelial neoplasia or suspicious features on initial biopsy) or in whom PSA concentration was persistently elevated. Tumours were staged using the TNM staging system. A central pathology review is also conducted.

### Selection of cases and controls

Cases were men aged 50–69 years, who underwent PSA measurement and had a histological diagnosis of primary prostate cancer. We defined localised cancer as T1–T2, NX or NO, MX or MO and advanced cancer as T3–T4 or N1 or M1; there were 1894 localised cases and 257 advanced cases (not taking account of the availability or not of anthropometric measurements). We defined high-grade cancer as Gleason grades 8–9; intermediate grade cancer as Gleason grade 7; and low-grade cancer as Gleason grade ⩽6.

All participants with no evidence of prostate cancer after PSA testing, DRE and/or biopsy were eligible to be controls. Controls were stratum matched to cases by age (5-year bands), and the primary care centres from which they were recruited. The index date for controls was the date of the prostate check clinic. Such matching automatically matches for calendar time, as prostate check clinics were completed sequentially. Detailed descriptions of ProtecT and the protocol for nested case–control selection are published elsewhere (Zuccolo *et al*, 2008).

### Exposure assessment

Obesity indicators were measured as well as self-reported. The measured weight value was taken at the prostate check clinic appointment by clinical staff. The weight was recorded, and it was explained to the participant that general health measurements were taken in order to examine the links with prostate cancer. The participant was weighed to the nearest 0.1 kg, and the measurement was noted by the clinical staff.

Participants were given a self-completion diet, health and lifestyle questionnaire at the clinic; this included questions on body size and weight in stones/pounds, height in feet/inches, and inside leg measurement in inches. They were also provided with a tape measure with which to take waist and hip measurements themselves and return it together with the questionnaire. Participants were instructed regarding the use and placement of the tape measure in order to make the measurements. Each tape measurement (in inches) was marked with a single line on the tape measure, labelled with the relevant letter (W or H) (measured value) and also recorded in the relevant questionnaire box by the participant (self-reported measurement).

We used a metric based on both available measurements, derived from the measured value if that was available and the self-reported value otherwise. BMI was derived from weight and height measurements as kg m^−2^; WHR was computed as waist circumference divided by hip circumference.

In November 2008, there were 2167 prostate cancer cases and 11 638 controls, randomly selected from ∼100 000 men not diagnosed with prostate cancer. The data composition of the stage and grade analyses is present in Table 1a. Data on BMI could be derived for 4769 controls and 1025 cases; data on WHR could be derived for 4917 controls and 1075 cases, whereas waist circumference data were available for 5020 controls and 1089 cases (Table 1a). Subjects included in the final BMI analyses had complete data on BMI, age and family history, and comprised 3931 controls and 919 cases. For the final analyses for waist circumference, 4156 controls and 960 cases had complete data on waist circumference, age and family history. For the final WHR analyses there were 4069 controls and 948 cases with complete data on WHR, age and family history (analyses presented in Table 2).

The study received ethical approval from Trent Multicentre Research and Ethics Committee and all participants provided written informed consent.

### Statistical analysis

BMI was categorised according to the WHO suggestions using categories <25.0, 25.0–29.9, ⩾30.0 in kg m^−2^ (Expert panel on the identification evaluation and treatment of overweight and obesity in adults, 1998). Waist circumference and WHR were categorised into tertiles based on the distribution of these measures amongst controls, as follows: waist circumference (⩽91.4, 91.5–99.1, >99.1 cm) and WHR (<0.91, 0.91–0.95, >0.95).

Conditional logistic regression was used to estimate odds ratios (ORs) and 95% confidence intervals (CIs) for associations of obesity with total prostate cancer. We computed two models. First, the basic conditional logistic regression model, in which the stratum matching of cases to controls by age and recruitment centre is taken into account. The second multivariable model was additionally adjusted for exact age at prostate check clinic and family history, as these are the established risk factors for prostate cancer.

The ORs for associations with advanced and localised cancer *vs* controls and for low, intermediate and high-grade disease *vs* controls were compared using a multinomial logistic regression model. This model provides a statistical test for heterogeneity in ORs comparing associations of the obesity indicators with localised *vs* advanced prostate cancers, but it is unconditional; it was, therefore, adjusted for exact age at prostate check clinic and the study centre where the recruiting general practice was based (nine-level variable).

The maximum number (1089 cases and 5020 controls) of subjects used in the analysis, before adjusting for other factors, was compared with the eligible participants not included in the analysis (1078 prostate cancer cases and 6618 randomly selected controls) using *t*-tests for continuous variables and the *χ*^2^-test for binary response variables.

### The PSA detection bias

Some previous studies have shown a decreased PSA level with increasing adiposity (Baillargeon *et al*, 2005; Werny *et al*, 2007). This suggests that there could be differential prostate cancer detection with respect to obesity, particularly for screen-detected case finding by PSA-based thresholds for biopsy. Therefore, to assess the potential for PSA detection bias, associations of the adiposity measures with serum PSA concentration amongst controls were investigated using linear regression performed on log-transformed PSA concentrations, adjusted for exact age and recruitment centre.

Analyses were conducted using Stata/IC 10.1 for Windows (StataCorp, 2007, College Station, TX, USA).

## Results

Table 1b presents the baseline characteristics of the maximum number of cases and controls with adiposity data (1089 cases and 5020 controls). A greater proportion of cases (8.1%) than controls (5.2%) reported a family history of prostate cancer in first degree relatives. In the highest tertiles, there were 18.4% cases and 21.5% controls with BMI ⩾30.0 kg m^−2^; the percentage of cases with >99 cm waist circumference was 30.5%, whereas that of controls was 32.1% 31.8% of cases and 33.9% of controls had a WHR >0.95.

The percentage of potentially eligible cases (2167) with missing BMI information was 52.7% the respective percentage for potentially eligible controls (11 638) was 59%. The percentages for missing data on waist measurements were 49.8% for cases and 56.9% for controls. For 50.4% of cases and 57.8% of controls information on WHR could not be derived. The maximum number (1089 cases and 5020 controls) of subjects used in the analysis, before adjusting for other factors, was similar to the eligible participants not included in the analysis (1078 prostate cancer cases and 6618 randomly selected controls) in terms of family history of prostate cancer in 1st degree relatives (*χ*^2^ *P*-value=0.100), but not in terms of age (*P*-value from *t*-test <0.001). Those included in the analysis were on average a year older than those not included.

Table 2 presents the association of the adiposity measures BMI, waist measurement and WHR with total prostate cancer. There was no evidence of any important relationship between any of the measures of adiposity and total prostate cancer.

To examine the association of the obesity measures with stage of prostate cancer, we calculated the ORs and CIs for the categories of BMI/waist circumference/WHR. These are shown in Table 3. For the fully adjusted model, that is, controlling for age, family history and recruitment centre, the smallest *P*-value for BMI was marginal and limited to localised prostate cancer (0.053). Results were similar for the basic model, adjusted for exact age and survey centre (not shown). Further data on age, family history and other medical conditions such as diabetes and cardiovascular disease are shown in the Supplementary Material.

Results in Table 4 are ORs for the categories of BMI, waist circumference and WHR, in order to examine the association of obesity with grade of prostate cancer. For the fully adjusted model for BMI, we observed a weak association with prostate cancer grade, limited to the low-grade group (*P*-trend 0.045; highest *vs* lowest tertile OR: 0.76, 95% CI: 0.59, 0.97), with the effect being small and the respective CI close to 1. Results for the basic model were largely similar and are not presented.

We did not observe any associations with either waist circumference or WHR after adjusting for age, family history and study centre for disease stage (all *P*>0.35) or grade (all *P*>0.16).

Amongst controls, the geometric mean PSA values for BMI categories (<25.0, 25.0–29.9, ⩾30.0 kg m^−2^) were 1.11 (1.07, 1.16), 1.04 (1.01, 1.07), 0.95 (0.90, 0.99) in ng ml^−1^, respectively (*P*-value <0.001 for highest *vs* lowest group). Defining L as the lowest group, M as the middle group and H as the highest group, the percent differences in means were between L and M 6.3%, between M and H 8.6%, and between L and H 14.4%. The geometric mean PSA values for waist measurement categories (<91.5, 91.5–99, >99 cm) were 1.08 (1.04, 1.11), 1.06 (1.02, 1.10), 0.96 (0.92, 1.00) in ng ml^−1^, respectively (*P*-value <0.001 for highest *vs* lowest group). The percent differences in means were between L and M 1.8%, between M and H 9.4%, and between L and H 11.1%. The geometric mean PSA values for WHR categories (<0.91, 0.91–0.95, >0.95) were 1.04 (1.00, 1.08), 1.06 (1.02, 1.10), 1.00 (0.96, 1.03) in ng ml^−1^, respectively (*P*-value 0.06, for highest *vs* lowest group). The percent differences in means were between L and M 1.9%, between M and H 5.6%, and between L and H 3.8%.

In a sensitivity analysis, we compared different sources of measurement for weight, waist and hip – as well as the derived WHR. Comparison was between measured and self-reported values. Measured and self-reported weight had a Spearman correlation coefficient of 0.98 (significance level 0.01). The correlation coefficient for measured and self-reported waist circumference was 0.99 (significance level 0.01). For measured and self-reported hip measurements the correlation coefficient was 0.98 (significance level 0.01). Therefore, it is unlikely that using different sources of measurement introduces bias.

## Discussion

We found no evidence that our measures of central adiposity were associated with PSA-detected prostate cancer. There was only weak evidence that general adiposity was associated with decreased risk of low-grade prostate cancer.

Most studies use BMI as a measure of obesity, although BMI cannot distinguish between lean and fat mass. This can be problematic for measurement and might account for contradictory results from several retrospective and prospective cohort studies using BMI to evaluate adiposity (Andersson *et al*, 1997; Nilsen and Vatten, 1999; Rodriguez *et al*, 2001; Calle *et al*, 2003; Giovannucci *et al*, 2003; Jonsson *et al*, 2003). Therefore, we used additional indicators, that is, waist measurements and WHR as estimates of central adiposity and excess abdominal fat (Arner, 1997). Measures of central adiposity are also better suited for measurements in middle-aged men. Abdominal obesity has been linked to several chronic conditions through mechanisms involving hormonal and metabolic changes (Kaaks and Stattin, 2010). However, very few studies (Giovannucci *et al*, 1997; Hsing *et al*, 2000; Lee *et al*, 2001; MacInnis *et al*, 2003; Hubbard *et al*, 2004; von Hafe *et al*, 2004; Wallstrom *et al*, 2009) have examined prostate cancer associations with abdominal adiposity; prospective cohort and case–control studies have shown increased risk with increasing WHR but no association with BMI (Hsing *et al*, 2000; von Hafe *et al*, 2004). This might imply that abdominal fat rather than general obesity may be associated with prostate cancer risk.

Associations reported in individual studies and for certain subgroups of men have not been seen consistently across studies. Obesity (as defined by BMI >30 kg m^−2^) has been linked with increased risk in some cohort studies (Lew and Garfinkel, 1979; Snowdon *et al*, 1984; Chyou *et al*, 1994), but not in some other cohort and case–control studies (Kolonel *et al*, 1988; Mills *et al*, 1989; Nomura and Kolonel, 1991; Kolonel, 1996; Andersson *et al*, 1997; Nomura, 2001; Friedenreich *et al*, 2004). Giovannucci *et al* (2003), in their prospective cohort study, reported inverse associations between BMI and prostate cancer risk in younger men or those with a family history. Rohrmann *et al* (2003) observed a reduced risk of high-grade disease in those with a family history, but an increased risk of high-grade disease in obese men <50 years old, in a case–control study. Two cohort studies of general (Engeland *et al*, 2003) and central (Wallstrom *et al*, 2009) obesity found increased risks in younger obese men.

Some hormonal and metabolic alterations that occur in obesity, such as decreased testosterone, may decrease prostate cancer risk; however, other alterations such as high insulin, insulin-like growth factor 1 (IGF-1) and/or leptin levels (Frystyk *et al*, 1995) have mitogenic effects (McKeehan *et al*, 1984) and can potentially increase risk by promoting prostate cancer progression (Yu and Rohan, 2000; Chan *et al*, 2002; Jenks, 2010). Testosterone is involved in prostatic growth and low levels prevent proliferation in the prostate; therefore, decreased testosterone in obese men may explain a protective effect of obesity against incident prostate cancer. At the same time, high testosterone helps maintain differentiation in prostatic epithelium, thus, preventing tumour progression, which means that the low testosterone levels in obese men increase the risk of tumours that are poorly differentiated/high grade. The IGF-1 is involved in androgen-independent progression of prostate cancer and leptin induces migration in prostate cancer cells, thus obesity-induced hormonal changes may promote tumour progression (Amling *et al*, 2004; Freedland *et al*, 2004). Leptin and insulin/IGF-1 are high in obese men (Hoda *et al*, 2010) and suppress androgen levels, moreover leptin has been implicated in advanced and high-grade prostate cancer in two case–control studies (Saglam *et al*, 2003; Ribeiro *et al*, 2006). Therefore, the simultaneous presence of high insulin and leptin levels and low testosterone levels explains the reduced risk of incident tumours, but the increased risk of progression in existing prostate cancer tumours.

One of the reasons why obesity has been hypothesised to be associated with greater risk of prostate cancer progression in case–control, cohort and prospective studies (Andersson *et al*, 1997; Putnam *et al*, 2000; MacInnis *et al*, 2003; Dal Maso *et al*, 2004; Baillargeon *et al*, 2005; Freedland *et al*, 2005; Wright *et al*, 2007; Gross *et al*, 2009), as opposed to initiation (Giovannucci *et al*, 2003), is that obese men tend to have higher oestradiol and lower testosterone levels, which have been associated with more advanced and poorly differentiated tumours (Hsing *et al*, 2002; Massengill *et al*, 2003; Platz *et al*, 2005) as explained above. This implies that different associations of obesity might be observed for prostate cancers of different stage or grade. Evidence from prospective studies is beginning to show that energy intake in excess of expenditure, captured by a higher BMI, may affect prostate carcinogenesis and, in particular, risk of advanced disease, and that energy imbalance may function late in the carcinogenic pathway, therefore, facilitating progression rather than initiation of prostate tumours (Rodriguez *et al*, 2007).

The differences in risk trends in previous studies, mentioned above, are possibly because of different methods used for assessing obesity, and to different disease mechanisms acting at different prostate cancer stages and grades (Gong *et al*, 2006; MacInnis and English, 2006; Littman *et al*, 2007; Rodriguez *et al*, 2007). Prostate cancer has a long natural history, and it might be that BMI in earlier life is more important for the development of prostate cancer than adult BMI, explaining the absence of strong associations with BMI in most studies including the present study.

We do not consider differences in PSA values by BMI to be an important source of bias. For BMI, as well as the other adiposity measures, there was weak evidence that PSA levels were linked to obesity, as the absolute difference between the geometric mean PSA values in the highest and lowest categories did not exceed 0.16 ng ml^−1^, and the maximum percent difference in means between BMI categories was 14.4%.

The strengths of the study are its large sample size and a well-characterised population. In addition, the collection of both self-reported and clinically measured values for the assessment of the adiposity indicators used allows assessment of bias from different sources of measurement. We found no such bias in this study. However, BMI values could be derived from only 47% of cases and 42% of the total population. Waist circumference and WHR measurements had similarly low percentages. For all obesity variables, the percentage of cases with available information was higher than the respective percentage of controls. The reductions in sample size may have affected the power of the study to detect effects, and it is theoretically possible that associations may have differed in those included in the analysis compared to those who could not be included (selection bias). These are two study limitations, although the study remains large. Our results do not support the hypothesis that obesity is involved in prostate cancer progression, although our study is limited to PSA-detected disease.

## Figures and Tables

**Table 1a:** Composition of BMI, waist circumference and WHR data

	**Derived BMI data**	**Available waist circumference data**	**Derived WHR data**
**Controls**	**4769**	**5020**	**4917**
*Cases stage and grade*			
*Stage*			
Localised cases	919	970	957
Advanced cases	100	111	110
Unstaged cases	6	8	8
			
*Grade*			
Low	691	736	723
Intermediate	272	288	287
High	57	59	59
Ungraded cases	5	6	6
			
**Subjects with complete data on each of BMI, waist circumference, WHR and factors (age, family history) in final stage and grade analyses**			
	**BMI + factors**	**Waist circumference + factors**	**WHR + factors**
**Controls**	**4368**	**4583**	**4490**
*Cases stage and grade*			
*Stage*			
Localised cases	843	882	870
Advanced cases	86	88	88
Grade			
Low	632	665	654
Intermediate	242	248	247
High	55	57	57

Abbreviations: BMI=body mass index; WHR=waist-to-hip ratio.

The derived metric is defined as the measured value if available and the self-reported value otherwise. BMI was derived from weight and height measurements as kg m^−2^; WHR was computed as waist circumference divided by hip circumference.

**Table 1b:** Baseline characteristics of cases and controls

	**Case**			**Control**		
**Variable**	***n***	**Mean (s.d.)**	**%**	***n***	**Mean (s.d.)**	**%**
*Age at prostate check clinic*	1089	62.3 (5.0)		5013	62.1 (5.0)	
Missing	0		0.0	7		0.1
						
*Height (m)*	1078	1.76 (0.07)		4976	1.76 (0.07)	
Missing	11		1.0	44		0.9
						
*Ethnicity*						
White	1055		96.9	4927		98.1
Other	2		1.1	48		1.0
Missing	22		2.0	45		0.9
						
*Social class*						
Working	345		31.7	1560		31.1
Intermediate	131		12.0	583		11.6
Professional	370		34.0	1722		34.3
Missing	243		22.3	1155		23.0
						
*Family history in first degree relatives*						
Yes	88		8.1	259		5.2
No	888		81.5	4330		86.2
Missing	113		10.4	431		8.6
						
*PSA at PCC* *(ng ml*^−*1*^)	1089	8.85 (28.1)		5020	1.40 (1.4)	
Missing	0		0.0	0		0.0
						
*Stage*						
Localised	970		89.1			
Advanced	111		10.2	—		—
Missing	8		0.7			
						
*Grade*						
Low	736		67.6			
Intermediate	288		26.4			
High	59		5.4	—		—
Missing	6		0.6			

Abbreviations: PCC=prostate check clinic; PSA=prostate-specific antigen.

**Table 2:** Association of obesity measures with prostate cancer

	**Case**	**Control**	**OR (CI) for basic model, significance** a	**Adjusted OR (CI)** b
*BMI (kg m*^−*2*^)				
<25.0	264	1080	1.00	1.00
25.0–29.9	481	1996	0.97 (0.82, 1.15)	0.98 (0.82, 1.16)
⩾30.0	174	855	0.82 (0.66, 1.02)	0.83 (0.67, 1.03)
Trend *P*-value			0.083	0.097
				
*Waist (cm)*				
⩽91.4	385	1615	1.00	1.00
91.5–99.1	286	1217	1.01 (0.85, 1.19)	1.01 (0.85, 1.20)
>99.1	289	1324	0.94 (0.79, 1.12)	0.94 (0.80, 1.12)
Trend *P*-value			0.496	0.517
				
*Waist/hip ratio*				
⩽0.908	318	1369	1.00	1.00
0.909–0.952	328	1312	1.09 (0.91, 1.30)	1.09 (0.91, 1.30)
>0.952	302	1388	0.92 (0.77, 1.10)	0.93 (0.77, 1.11)
Trend *P*-value			0.368	0.395

Abbreviations: BMI=body mass index; CI=confidence interval; OR=odds ratio.

a Taking account of the matching variables age (in 5-year bands) and recruitment centre.

b Additional adjustment for age and family history.

BMI groupings according to the World Health Organisation suggested categorisations.

Ethnicity not controlled for because of the very small percentage (1%) of non-white participants.

**Table 3:** Associations of BMI, waist circumference and WHR with prostate cancer stage

	**Reference group**	**Stage**				
	**Controls**	**Localised**	**OR (CI)**	**Advanced**	**OR (CI)**	***P* for heterogeneity**
*BMI*						
<25.0	1178	239	1.00	27	1.00	
25.0–29.9	2219	448	1.00 (0.84–1.19)	39	0.77 (0.47–1.27)	
⩾30.0	971	156	0.79 (0.63–0.98)	20	0.94 (0.52–1.69)	
Trend *P*-value			0.053		0.745	0.74
						
*Waist (cm)*						
⩽91.4	1781	352	1.00	36	1.00	
91.5–99.1	1337	265	1.00 (0.84–1.19)	25	0.90 (0.54–1.51)	
>99.1	1465	265	0.91 (0.77–1.09)	27	0.89 (0.54–1.48)	
Trend *P*-value			0.355		0.585	0.82
						
*WHR*						
⩽0.908	1514	291	1.00	28	1.00	
0.909–0.952	1437	303	1.10 (0.92–1.31)	28	1.06 (0.62–1.80)	
>0.952	1539	276	0.93 (0.78–1.11)	32	1.14 (0.68–1.90)	
Trend *P*-value			0.473		0.681	0.53

Abbreviations: BMI=body mass index; CI=confidence interval; OR=odds ratio; WHR=waist-to-hip ratio.

Multinomial logistic regression model adjusted for age, survey centre, family history. All expected cell frequencies >5.

Groupings according to the WHO categorisation for BMI, and according to the distribution of controls for waist and WHR.

**Table 4:** Associations of BMI, waist circumference and WHR with grade of prostate cancer (high: Gleason grades 8–9; intermediate: Gleason grade 7; low: Gleason grade ⩽6)

	**Reference group**	**Grade**						
	**Controls**	**Low**	**OR (CI)**	**Intermediate**	**OR (CI)**	**High**	**OR (CI)**	***P* for heterogeneity**
*BMI*								
<25.0	1178	184	1.00	67	1.00	15	1.00	
25.0–29.9	2219	331	0.95 (0.78–1.16)	129	1.04 (0.76–1.40)	27	0.99 (0.52–1.87)	
⩾30.0	971	117	0.76 (0.59–0.97)	46	0.85 (0.58–1.25)	13	1.12 (0.53–2.38)	
Trend *P*-value			0.045		0.444		0.822	0.67
								
*Waist (cm)*								
⩽91.4	1781	270	1.00	95	1.00	22	1.00	
91.5–99.1	1337	199	0.98 (0.81–1.20)	74	1.02 (0.75–1.40)	17	0.99 (0.52–1.88)	
>99.1	1465	196	0.88 (0.72–1.07)	79	1.00 (0.74–1.36)	18	0.96 (0.51–1.80)	
Trend *P*-value			0.254		0.989		0.909	0.81
								
*WHR*								
⩽0.908	1514	212	1.00	86	1.00	23	1.00	
0.909–0.952	1437	222	1.10 (0.90–1.35)	89	1.11 (0.82–1.51)	19	0.85 (0.46–1.58)	
>0.952	1539	220	1.01 (0.83–1.24)	72	0.84 (0.61–1.16)	15	0.64 (0.33–1.23)	
Trend *P*-value			0.812		0.241		0.169	0.24

Abbreviations: BMI=body mass index; CI=confidence interval; OR=odds ratio; WHR=waist-to-hip ratio.

Multinomial logistic regression model adjusted for age, survey centre, family history. All expected cell frequencies >5.

Groupings according to the World Health Organisation categorisation for BMI, and according to the distribution of controls for waist and WHR.
