# Supplementary material for: Association of obesity with prostate cancer: a case-control study within the population-based PSA testing phase of the ProtecT study
Source: Br J Cancer. 2011 Jan 25;104(5):875–81. doi: 10.1038/sj.bjc.6606066 (PMC3048201; doi:10.1038/sj.bjc.6606066)
Supplement: Supplementary Material [file 6606066x1.doc]

| **Variable** | **Case** | **Control** | **Adjusted* OR(CI)** | **significance** |
| --- | --- | --- | --- | --- |
| **Age**  <=60.0 yrs  60.1-65.0 yrs  >65.0 yrs | 747  618  544 | 4,071  3,412  2,786 | 1.00  1.21(0.98,1.51)  1.43(1.06,1.94) | 0.082  0.020 |
| **p trend** |  |  |  | 0.020 |
| **Social class**  Working  Intermediate  Manag-prof/nal | 500  219  629 | 2,702  1,102  3,182 | 1.00  1.08(0.90,1.29)  1.11(0.97,1.28) | 0.388  0.138 |
| **p trend** |  |  |  | 0.140 |
| **Family history in 1^st^ d.rel.**  yes  no | 162  1,747 | 552  9,717 | 1.66(1.38,2.00)  1.00 |  |
| **p trend** |  |  |  | <0.001 |
| **Height**  <=68.0 inches  68.1-70.0 inch  >70.0 inches | 354  307  307 | 1,632  1,328  1,253 | 1.00  1.08(0.91,1.28)  1.13(0.95,1.34) | 0.381  0.170 |
| **p trend** |  |  |  | 0.158 |
| **Exercise** |  |  |  |  |
| Strenuous  No  Once/week  >once/week | 472  85  238 | 1,734  380  1,039 | 1.00  0.81(0.62,1.05)  0.82(0.68,0.98) | 0.114  0.031 |
| **p trend** |  |  |  | 0.024 |
| Moderate  <= twice/week  2-5 times/week  >5 times/week | 413  261  279 | 1,679  1,150  1,118 | 1.00  0.91(0.76,1.09)  1.00(0.84,1.20) | 0.309  0.995 |
| **p trend** |  |  |  | .909 |
| Mild  No  1-5 times/week  >5 times/week | 303  220  237 | 1,011  967  918 | 1.00  0.73(0.60,0.90)  0.87(0.71,1.05) | 0.002  0.152 |
| **p trend** |  |  |  | 0.114 |
| Weekly vigorous exercise  None  1-2 times  3-4 times  5+ times | 492  377  173  70 | 2,105  1,682  681  334 | 1.00  0.95(.82,1.11)  1.06(.87,1.29)  0.87(.66,1.15) | .546  .558  .336 |
| **p trend** |  |  |  | .701 |
| **Medical conditions** |  |  |  |  |
| TypeII diabetes  Yes  no | 68  972 | 370  4,145 | 0.81 (0.61,1.06)  1.00 |  |
| **p trend** |  |  |  | 0.120 |
| **Variable** | **Case** | **Control** | **Adjusted* OR(CI)** | **significance** |
| Age at diabetes (>=30 yrs)  <=55.0 yrs  55.1-60.0 yrs  >60.1 yrs | 12  14  15 | 26  17  29 | 1.00  2.02(0.73,5.58)  1.88(0.55,6.38) | 0.173  0.314 |
| **p trend** |  |  |  | 0.543 |
| Diabetes medication  Yes  no | 28  618 | 134  2,074 | 0.73 (0.48,1.12)  1.00 |  |
| **p trend** |  |  |  | 0.154 |
| Angina  Yes  No | 59  977 | 328  4,157 | 0.78 (0.58,1.04)  1.00 |  |
| **p trend** |  |  |  | 0.092 |
| Age angina started  <=52.0 yrs  52.1-59.0 yrs  >59.0 yrs | 12  16  8 | 20  29  18 | 1.00  1.13(0.43,2.98)  0.91(0.27,3.09) | 0.800  0.878 |
| **p trend** |  |  |  | 0.882 |
| Stroke  Yes  no | 20  1,016 | 102  4,393 | 0.86(0.52,1.41)  1.00 |  |
| **p trend** |  |  |  | 0.511 |
| Age at stroke  <=54.0 yrs  54.1-60.0 yrs  >60.0 yrs | 3  2  2 | 5  1  1 | Unreliable, all  expected cell frequencies <5 |  |
| **p trend** |  |  |  |  |
| Myocardial infarction  Yes  no | 40  1,004 | 235  4,287 | 0.74 (0.52,1.05)  1.00 |  |
| **p trend** |  |  |  | 0.087 |
| Age at MI  <=50.0 yrs  50.1-57.0 yrs  >57.0 yrs | 5  7  4 | 11  11  9 | Unreliable estimates, some  expected cell frequencies <5 |  |
| **p trend** |  |  |  |  |
| **Smoking** |  |  |  |  |
| Smoked now/past  Never smoked | 737  400 | 3,307  1,624 | 0.92 (0.80,1.06)  1.00 |  |
| **p trend** |  |  |  | 0.229 |
| **Age started smoking**  <=16.0 yrs  16.1-18.0 yrs  >18.0 yrs | 276  169  157 | 1,170  668  602 | 1.00  1.09(0.87,1.36)  1.14(0.91,1.43) | 0.420  0.229 |
| **p trend** |  |  |  | 0.226 |
| **Current smoker**  Yes  No | 141  562 | 549  2,321 | 1.02 (0.83,1.26)  1.00 |  |
| **p trend** |  |  |  | 0.819 |

| **Variable** | **Case** | **Control** | **Adjusted* OR(CI)** | **significance** |
| --- | --- | --- | --- | --- |
| **CURRENT USE** |  |  |  |  |
| **Cigarettes/day**  Under 10 a day  10 to 19 a day  >=20 a day | 36  29  21 | 51  69  42 | 1.00  0.66(0.35,1.25)  0.76(0.36,1.61) | 0.204  0.477 |
| **p trend** |  |  |  | 0.419 |
| **Cigars/week**  None  <=3  >3 | 27  0  20 | 37  7  26 | Unreliable,some expected cell frequencies <5 |  |
| **p trend** |  |  |  |  |
| **PAST USE** |  |  |  |  |
| **Cigarettes/day**  Under 10 a day  10 to 19 a day  >=20 a day | 132  157  160 | 442  584  662 | 1.00  0.96(0.73,1.25)  0.85(0.65,1.10) | 0.749  0.215 |
| **p trend** |  |  |  | 0.198 |
| **Cigars/week**  None  <=1  >1 | 121  5  54 | 281  10  137 | Unreliable,some expected cell frequencies <5 |  |
| **p trend** |  |  |  |  |
| **Age stopped smoking**  <=33.0 yrs  33.1-45.0 yrs  >45.0 yrs | 162  151  164 | 644  573  586 | 1.00  1.09(0.85,1.41)  1.15(0.89,1.49) | 0.497  0.278 |
| **p trend** |  |  |  | 0.322 |
| **Passive smoker**  Yes  no | 482  479 | 2,043  2,106 | 1.02 (0.89,1.18)  1.00 |  |
| **p trend** |  |  |  | 0.744 |

*groupings according to the distribution of controls*

**adjusted for the established factors for prostate cancer: age, family history*
